# Relação entre a Razão Nitrogênio Ureico/Creatinina e Prognóstico de Insuficiência Cardíaca em Todo o Espectro da Fração de Ejeção

**DOI:** 10.36660/abc.20220427

**Published:** 2023-03-20

**Authors:** Yuan Kang, Conglin Wang, Xiaojing Niu, Zhijing Shi, Mingxue Li, Jianli Tian

**Affiliations:** 1 Department of Geriatrics Tianjin Medical University General Hospital Tianjin China Department of Geriatrics, Tianjin Medical University General Hospital, Tianjin – China

**Keywords:** Insuficiência Cardíaca, Nitrogênio da Ureia Sanguínea, Volume Sistólico, Prognóstico

## Abstract

**Fundamento:**

Em pacientes com insuficiência cardíaca (IC), devido à relativa deficiência do volume sanguíneo, a ativação do sistema neuro-hormonal leva à vasoconstrição renal, que afeta o teor de nitrogênio ureico (NU) e creatinina (C) no organismo, sendo que NU e C são facilmente afetados por outros fatores. Portanto, a razão NU/C pode ser utilizada como mais um marcador para o prognóstico da IC.

**Objetivo:**

Explorar o prognóstico do desfecho adverso da IC no grupo NU/C alta em comparação com o grupo NU/C baixa em todo o espectro da fração de ejeção.

**Métodos:**

De 2014 a 2016, pacientes sintomáticos hospitalizados com IC foram recrutados e acompanhados para observar desfechos cardiovasculares adversos. Foram realizadas análise logística e a análise COX para determinar a significância. Valores de p<0,05 foram considerados estatisticamente significativos.

**Resultados:**

Na análise de regressão logística univariada, o grupo NU/C alta apresentou maior risco de desfecho adverso na insuficiência cardíaca com fração de ejeção reduzida (ICFEr) e insuficiência cardíaca com fração de ejeção preservada (ICFEp). A análise de regressão logística multivariada mostrou que o risco de morte cardíaca no grupo ICFEr foi maior do que no grupo NU/C baixa, enquanto o risco de morte por todas as causas foi significativo apenas em 3 meses (p<0,05) (Ilustração Central). O risco de morte por todas as causas no grupo NU/C alta no grupo ICFEP foi significativamente maior do que no grupo NU/C baixa em dois anos.

**Conclusão:**

O grupo NU/C alta está relacionado ao risco de mau prognóstico da ICFEP, não sendo inferior ao valor preditivo da fração de ejeção do ventrículo esquerdo (FEVE).

## Introdução

Nos últimos tempos, a insuficiência cardíaca (IC) tem sido frequentemente encontrada na população geriátrica. De acordo com as diretrizes de 2021 da European Heart Association, os pacientes com IC são classificados em (1) Insuficiência cardíaca com fração de ejeção reduzida (ICFEr), onde a fração de ejeção do ventrículo esquerdo (FEVE) reduzida é definida como ≤40%; (2) Insuficiência cardíaca com fração de ejeção na faixa média (ICFEfm), incluindo pacientes com FEVE entre 41% e 49%; e (3) Insuficiência cardíaca com fração de ejeção preservada (ICFEp), em que os pacientes apresentam FEVE ≥49%.^1^Embora novas estratégias sejam continuamente introduzidas para combater a IC, ela se mantém como um dos problemas com maiores taxas de mortalidade e reinternação entre os pacientes hospitalizados. Alguns estudos documentaram a associação da mortalidade de pacientes com IC com a falta de volume sanguíneo efetivo,^2^ mas outros estudos sugeriram que pacientes com IC teriam ativação excessiva de diferentes neuro-hormônios, como o sistema renina-angiotensina-aldosterona (RAAS) e sistema nervoso simpático (SNS), resultando em congestão venosa e insuficiência renal. Os mecanismos acima terão certo impacto no prognóstico.^3^

Em pacientes com IC, a insuficiência renal também é atribuída à diminuição da contratilidade miocárdica.^4^ Além de diminuir a taxa de filtração glomerular estimada (TFGe),^5^ a renina também aumenta a reabsorção de água e sal, levando ao aumento do nitrogênio ureico (NU).^6^ Portanto, a creatinina sérica (C) e o NU são considerados indicadores clínicos eficazes de mau prognóstico. Sob condições fisiológicas, o NU pode ser filtrado livremente no glomérulo, mas entre 30% e 40% é reabsorvido no túbulo renal.^5 , 7^ A reabsorção do NU também aumenta devido à ativação excessiva de neuro-hormônios em pacientes com IC,^8^ enquanto a ingestão de proteínas, aumento do catabolismo e outros fatores também alteram os níveis de NU.^9^ A C pode ser filtrada livremente no glomérulo, embora não seja reabsorvida no túbulo renal. A C também é facilmente afetada pela alimentação e outros fatores.^10 , 11^ Portanto, a razão NU/C pode ser um indicador de disfunção renal e uma medida do neuro-hormônio e da atividade nervosa simpática. Além disso, a razão também está relacionada a eventos adversos em pacientes com IC.^6^

Embora se desconheça a razão NU/C normal, estudos anteriores indicam que NU/C>25,5 é um fator de risco independente para predizer óbito em pacientes com IC aguda ou crônica.^7^ O NU é visto como um reflexo e diminuição do débito cardíaco. O NU é proporcional ao estado hemodinâmico do marcador prognóstico danificado.^9^ Sabe-se que o débito cardíaco é diverso em pacientes com IC em todo o espectro da fração de ejeção. No entanto, não há descrição da capacidade preditiva da razão NU/C em pacientes com IC em todo o espectro da fração de ejeção. Portanto, o presente estudo compara o prognóstico da IC em todo o espectro da fração de ejeção pela razão NU/C na admissão.

## Métodos

### População do estudo

O presente estudo observacional retrospectivo foi realizado em 2.255 pacientes sintomáticos com IC que passaram pelo serviço ambulatorial do Hospital Geral da Universidade de Medicina de Tianjin e do Hospital Torácico de Tianjin entre fevereiro de 2014 a junho de 2016 em Tianjin, China. Critérios de inclusão: (1) Pacientes ≥18 anos com IC sintomática (classe funcional III–IV da NYHA); (2) NU, C e outros índices laboratoriais estimados nas primeiras 24 horas de internação. Critérios de exclusão: (1) Pacientes com indicadores incompletos; (2) Pacientes sem informação prognóstica; (3) Pacientes com tuberculose severa ou tumor maligno. A IC foi diagnosticada seguindo as diretrizes da European Society of Cardiology (ESC) de 2021 e foi examinada por pelo menos dois médicos com taxas de frequência, com base nos sintomas, sinais, resultados laboratoriais e avaliação da função cardíaca do paciente. Todos os pacientes assinaram o termo de consentimento informado para participar do estudo. O programa de pesquisa está de acordo com os princípios da Declaração de Helsinque e aprovado pelo Comitê de Ética Médica do Hospital Geral da Universidade de Medicina de Tianjin (Aprovação do Comitê de Ética nº: IRB2017.029–01, registro no.: CHICCTR-ERC-17011820).

### Informações preliminares e exames laboratoriais

Para todos os indivíduos, informações demográficas gerais, comorbidades, medicação, dados de ecocardiografia e resultados laboratoriais foram registrados. O diagnóstico de IC e o agrupamento de acordo com a fração de ejeção se basearam nas diretrizes da *European Society of Cardiology* (ESC) de 2016.^12^ Como a maioria dos pacientes não foi admitida com o estômago vazio, o sangue venoso periférico foi coletado no segundo dia de internação e os índices laboratoriais estimados. Todos os pacientes foram submetidos à ecocardiografia Doppler colorida transtorácica dentro de 48 horas após a internação. Os instrumentos utilizados são equipamentos clínicos padrão internacionalmente reconhecidos e os resultados do ultrassom foram avaliados por médicos com formação profissional.

### População do estudo: Seguimento e desfechos do estudo

Os desfechos deste estudo foram definidos como readmissão por IC, morte cardíaca e morte por todas as causas, e o prazo para o resultado clínico foi de 3 meses, 12 meses e 24 meses, respectivamente. O presente estudo foi acompanhado por 2 anos por meio de consultas ambulatoriais ou comunicação telefônica. As equipes de seguimento foram compostas por médicos treinados. Os médicos realizaram a coleta de dados e o seguimento de todos os pacientes inscritos no grupo por meio de questionários impressos. A versão impressa dos prontuários médicos foi arquivada. Após a conclusão do seguimento, dois médicos selecionaram 10% dos casos dos pacientes do grupo para verificação de dados, incluindo verificações de questionários impressos e seguimento por telefone. Dos 2.099 pacientes, todos concluíram seguimento de dois anos sem nenhuma intervenção, e os pesquisadores obtiveram informações prognósticas.

### Análise estatística

De acordo com as diferentes frações de ejeção, 2.099 indivíduos foram divididos em três grupos e, de acordo com o nível de NU/C, os indivíduos foram divididos em dois subgrupos (NU/C≤25,5 e NU/C>25,5). Primeiramente, para testar a normalidade da distribuição dos dados, utilizou-se o teste de Kolmogorov-Smirnov. Para variáveis contínuas, utilizou-se o teste T independente ou o teste da soma de postos de Wilcoxon, em que as variáveis que satisfizeram a distribuição normal foram representadas por média±desvio padrão, enquanto a mediana e o intervalo interquartil foram usados para representar as variáveis com distribuição não normal. As variáveis categóricas são expressas como números absolutos e porcentagens. Utilizou-se o teste do qui-quadrado de Pearson ou o teste exato de Fisher para comparar as razões entre os grupos e foram obtidas as características basais dos indivíduos. Posteriormente, empregou-se a análise de regressão logística para comparar o prognóstico de 3 meses, 12 meses e 24 meses em diferentes grupos NU/C. Foram usadas as variáveis demográficas e comorbidades significativas encontradas na IC em todo o espectro da fração de ejeção na análise univariada para ajustar fatores de confusão, incluindo idade, sexo, etilismo, diabetes, infarto do miocárdio, arritmia, infecção pulmonar, anemia, etc. Os resultados foram expressos por *odds ratio* (OR) e intervalo de confiança (IC) de 95%. De acordo com os diferentes grupos de fração de ejeção, ajustamos os fatores demográficos significativos comuns e os fatores de confusão dos indicadores laboratoriais, como sexo, idade, NU, C, hemoglobina e assim por diante. Tendo como referência o grupo de baixo NU/C (NU/C>25,5), utilizou-se a regressão logística para analisar a mortalidade por todas as causas em dois anos. A análise da curva ROC ajudou a avaliar a predição do grupo NU/C de mortalidade por todas as causas em dois anos. Além disso, de acordo com o ponto de corte da curva ROC (ponto de corte=20,4043), dividiu-se NU/C em um novo grupo para comparação das características basais relacionadas e os fatores prognósticos (razão NU/C elevada*≤20,4043, razão NU/C baixa*>20,4043). Todas as medidas foram bilaterais e p<0,05 foi considerado estatisticamente significativo. Todas as análises estatísticas foram realizadas pelo software estatístico SPSS (versão 22.0) IBM Corp.

## Resultado

### Características clínicas

Um total de 124 pacientes sem informações de NU ou C e 31 pacientes com tuberculose severa ou tumores malignos foram excluídos, e os restantes 2.099 pacientes sintomáticos com IC foram incluídos neste estudo. A idade média dos 2.099 pacientes incluídos neste estudo foi de 70 (61–79), dos quais 794 eram mulheres (37,8%). A C média nesta população foi de 105,4±64,3 mg/dL, e o NU médio foi de 9,5±23,8 mmol/L ( Tabela 1 , tabela suplementar 1).

**Tabela 1:** – Características basais de diferentes grupos de fração de ejeção com IC sintomática

	Total	ICFEr		Valor de p	ICFEfm		Valor de p	ICFEp		Valor de p
			
Características clínicas		Razão NU/C baixa	Razão NU/C elevada		Razão NU/C baixa	Razão NU/C elevada		Razão NU/C baixa	Razão NU/C elevada	
Idade	70 (61–79)	66 (58–76)	70 (61–79)	0,004	72 (62–80)	74 (63–80)	0,369	74 (64–81)	72 (63–82)	0,820
Mulheres	794 (37,8)	299 (37,6)	88 (58,3)	<0,001	193 (33,6)	57 (44,5)	0,013	109 (32,3)	48 (43,2)	0,023
Tabagismo	288 (13,8)	132 (16,8)	20 (13,2)	0,168	237 (41,3)	53 (41,7)	0,502	115 (34,1)	26 (23,4)	0,022
Etilismo	849 (40,6)	368 (46,5)	50 (33,1)	0,001	79 (13,8)	13 (10,2)	0,176	38 (11,3)	6 (5,4)	0,046
**Comorbidades**										
Hipertensão	1235 (58,8)	507 (63,8)	95 (62,9)	0,454	332 (57,7)	73 (57,0)	0,480	184 (54,3)	44 (39,6)	0,005
FA	578 (27,5)	152 (19,1)	39 (25,8)	0,041	161 (28,0)	54 (42,2)	0,001	122 (36,0)	50 (45)	0,056
Diabetes	646 (30,8)	266 (33,5)	74 (49,0)	<0,001	161 (28,0)	36 (28,1)	0,528	76 (22,4)	33 (29,7)	0,077
IAM	951 (45,3)	412 (51,8)	63 (41,7)	0,014	276 (48,5)	44 (34,4)	0,003	128 (37,8)	28 (25,2)	0,010
DAC	1668 (79,5)	648 (81,5)	123 (81,5)	0,533	467 (81,2)	95 (74,2)	0,050	256 (75,5)	79 (71,2)	0,215
Arritmia	968 (46,1)	319 (40,1)	76 (50,3)	0,013	275 (47,8)	75 (58,6)	0,017	161 (47,5)	62 (55,9)	0,078
Insuficiência renal	423 (20,2)	160 (20,1)	38 (25,2)	0,101	102 (17,7)	29 (22,7)	0,123	68 (20,1)	26 (23,4)	0,264
Infecção pulmonar	575 (27,4)	163 (20,5)	43 (28,5)	0,021	161 (28,0)	53 (51,4)	0,002	114 (33,6)	41 (36,9)	0,299
Anemia	310 (14,8)	86 (10,8)	31 (20,5)	0,001	75 (13,0)	25 (19,5)	0,042	66 (19,5)	27 (24,3)	0,168
**Exames laboratoriais**										
NT-ProBNP (pg/mL)	6300,1± 7467,7	5219,2± 6419,3	7931,8± 9352,9	<0,001	6352,7± 7504,5	8990,8± 8875,4	0,002	8990,8± 8875,4	8990,8± 8875,4	0,501
Lactato desidrogenase (U/L)	331,0± 344,1	336,6± 12,3	309,8± 247,3	0,035	329,0± 288,3	357,2± 416,5	0,183	340,0± 468,3	271,8± 146,37	0,011
Aspartato aminotransferase (U/L)	23,2 (16,5–43,2)	22 (16–45,9)	23,5 (16–44,5)	0,942	24 (17–45,7)	25 (16,8–44,6)	0,820	18 (12–33,1)	17 (11,7–38,3)	0,780
Creatinina sérica (mg/dL)	105,4±64,3	111,7±72,9	86,7±41,5	0,004	107±58,1	87,6±18,3	<0,001	109,3±69,7	86,7±49,53	0,046
Nitrogênio ureico (mmol/L)	9,5±23,8	7,3±4,0	15,4±25,5	<0,001	7,3±3,7	16,1±43	<0,001	7,7±4,7	26,2±84,4	<0,001
Ácido úrico (umol/L)	386,5± 150,4	386,5± 133,5	432,9± 177,3	<0,001	367,4± 142,1	441,9± 192,8	<0,001	372,5± 148,1	402,2± 187,9	0,038
Hemoglobina (g/L)	127 (111–142)	131 (116–146)	126 (108,3–141)	0,034	127 (112,2–142)	122,5 (105–141)	0,439	123 (106–137)	118 (105,5–135,5)	0,455
Distribuição de hemácias	13 (12,3–14,1)	12,9 (12,1–13,7)	13,2 (12,4–14,7)	<0,001	13 (12,2–14,1)	13,4 (12,7–15)	0,002	13,2 (12,3–14,3)	14 (13–15,2)	<0,001
Hemácias (*10^12^/L)		4,4±0,8	4,3±0,9	0,153	4,3±0,7	4,4±1,2	<0,001	4,1±0,7	3,9±0,8	0,586
Volume específico de hemácias	55,9±25,5	53,4±23,2	57,1±26,5	<0,001	53,6±24,2	55,3±27,1	0,034	61,5±27,5	64,9±29,9	0,006
**Histórico de medicamentos**										
IECA	709 (33,8)	320 (40,3)	51 (33,8)	0,080	189 (32,9)	31(24,2)	0,034	94 (27,7)	24 (21,6)	0,125
BRA	511 (24,3)	213 (26,8)	32 (21,2)	0,089	141 (24,5)	32(25,0)	0,495	74 (21,8)	19 (17,1)	0,177
Betabloqueadores	1323 (63,0)	567 (71,3)	91 (60,3)	0,005	363 (63,1)	70(54,7)	0,048	172 (50,7)	60 (54,1)	0,310
Diuréticos	1367 (65,1)	503 (63,3)	109 (72,2)	0,021	381 (66,3)	87(68,0)	0,397	216 (63,7)	71 (64,0)	0,529
Digitálicos	575 (27,4)	192 (24,2)	53 (35,1)	0,004	143 (24,9)	39(30,5)	0,117	107 (31,6)	41 (36,9)	0,176
**Ecocardiograma com doppler colorido**										
AE (mm)	42,5±7,6	41,9±6,9	41,6±7,3	0,915	42,9±7,1	43,4±7,8	0,141	42,9±8,3	42,8±10,9	0,002
VE (mm)	56,4±10,3	56,8±10,1	56,2±10,1	0,116	56,6±9,9	56,3±11,3	0,020	56,0±10,5	53,9±12,1	0,090
AD (mm)	39,3±8,8	37,1±6,8	40,2±8,4	0,038	39,2±8,3	56,3±11,3	0,056	41,1±9,9	41,9±11,3	0,109
VD (mm)	24 (18–30)	23 (16,5–32)	23 (18,1–31,2)	0,723	22 (18,2–30,6)	23 (17,4–31,9)	0,686	28 (20,2–32,5)	28 (20,3–34,5)	0,698

*ICFEr: insuficiência cardíaca com fração de ejeção reduzida; ICFEfm: Insuficiência cardíaca com fração de ejeção na faixa média; ICFEp: insuficiência cardíaca com fração de ejeção preservada; NU/C: nitrogênio ureico e creatinina; FA: fibrilação atrial, IAM: infarto agudo do miocárdio; DAC: doença arterial coronariana; IECA: inibidores da enzima conversora de angiotensina; BRA: bloqueadores de receptor de angiotensina; AE: átrio esquerdo; VE: ventrículo esquerdo; AD: átrio direito; VD: ventrículo direito.*

### Análise de sobrevida

**Figura 1:**
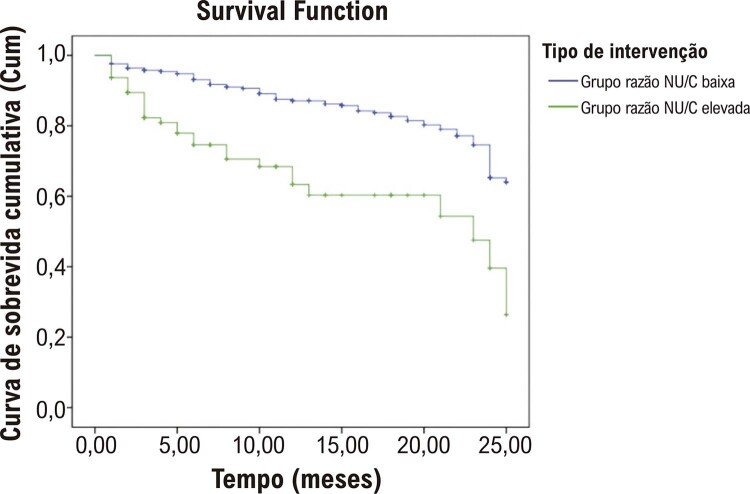
– Curvas de sobrevida de Kaplan–Meier em pacientes com razões NU/C baixas e elevadas. NU/C: nitrogênio ureico e creatinina.

Na análise de regressão logística do grupo ICFEr com variáveis não ajustadas, em comparação com o grupo razão NU/C baixa, o risco de morte cardíaca no grupo razão NU/C elevada foi maior do que no grupo razão NU/C baixa em 3 meses, 12 meses e 24 meses, e o risco de morte por todas as causas em 3 meses foi 2,062 vezes maior do que no grupo razão NU/C baixa ( Tabela 2 ). No grupo ICFEfm, não houve diferença significativa no resultado clínico em cada ponto de seguimento. No grupo ICFEP, o risco de reinternação por IC no grupo razão NU/C elevada foi maior do que no grupo razão NU/C baixa no 12º e 24º meses de seguimento, e o risco de morte por todas as causas foi 2,1 vezes maior do que no grupo razão NU/C baixa (p<0,005). Por meio da análise de regressão logística, constatou-se que, após ajustar os fatores de confusão correspondentes, o risco de morte cardíaca e morte por todas as causas em 3 meses em cada ponto de seguimento no grupo ICFEr ainda era maior do que no grupo razão NU/C baixa, e o risco de reinternação por IC e morte por todas as causas no grupo ICFEP ainda era significativamente maior do que no grupo razão NU/C baixa aos 12 e 24 meses ( Ilustração Central , tabela suplementar 2). Para toda a população com IC, o risco de morte por todas as causas no grupo razão NU/C elevada foi significativamente maior do que no grupo razão NU/C baixa no grupo ICFEP. Nos grupos ICFEr e ICFEp, em comparação com o grupo razão NU/C baixa, o grupo NU/C elevada apresentou uma taxa de mortalidade por todas as causas significativamente maior em dois anos após o ajuste para fatores mistos ( Tabela 3 ). No grupo ICFEP, essa tendência significativa também foi observada na análise de Cox, em que o risco do grupo razão NU/C elevada foi 3,280 vezes maior do que o do grupo razão NU/C baixa após ajuste para riscos relacionados (p<0,001, Tabela 4 ). As curvas de sobrevida de Kaplan–Meier no grupo ICFEP com razões NU/C se encontram na figura 1 (p<0,001).

**Tabela 2:** – FCs ajustadas (IC 95%) de reinternação por IC/morte cardíaca/morte por todas as causas em pacientes com IC com NU/C baixa vs. NU/C elevada

Prognóstico clínico	ICFEr	Valor de p	ICFEfm	Valor de p	ICFEp	Valor de p
		
	OR ajustada (IC 95%)		OR ajustada (IC 95%)		OR ajustada (IC 95%)	
**Reinternação por IC**						
3 meses	2,234 (0,932–5,351)	0,071	1,346 (0,519–3,493)	0,541	2,058 (0,872–4,855)	0,099
12 meses	0,790 (0,465–1,343)	0,384	0,892 (0,529–1,506)	0,67	1,849 (1,103–3,101)	0,02
24 meses	0,711 (0,429–1,177)	0,185	0,948 (0,582–1,544)	0,829	1,784 (1,087–2,929)	0,022
**Morte cardíaca**						
3 meses	2,508 (1,075–5,850)	0,033	0,456 (0,128–1,624)	0,226	1,091 (0,426–2,794)	0,855
12 meses	1,972 (1,118–3,480)	0,019	0,766 (0,397–1,480)	0,427	1,225 (0,656–2,288)	0,524
24 meses	2,062 (1,287–3,301)	0,003	1,046 (0,623–1,758)	0,864	1,095 (0,644–1,860)	0,738
**Morte por todas as causas**						
3 meses	2,608 (1,221–5,572)	0,013	0,748 (0,269–2,075)	0,577	1,717 (0,768–3,838)	0,188
12 meses	1,543 (0,863–2,761)	0,144	1,098 (0,623–1,935)	0,747	2,101 (1,227–3,598)	0,007

*Ajustado para sexo, idade, NU, C, hemoglobina. ICFEr: insuficiência cardíaca com fração de ejeção reduzida; ICFEfm: Insuficiência cardíaca com fração de ejeção na faixa média; ICFEp: insuficiência cardíaca com fração de ejeção preservada.*

**Figura Central:**
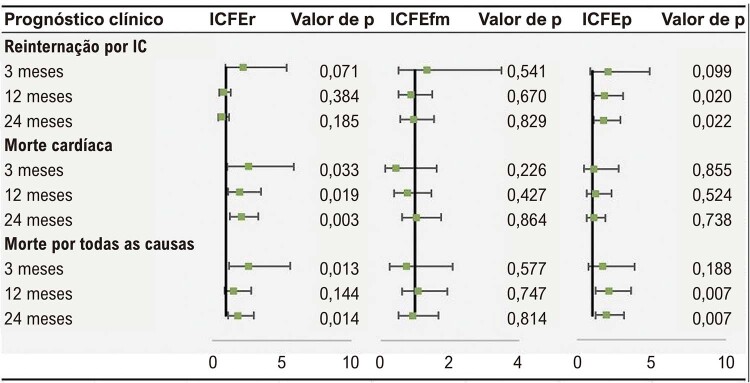
: Relação entre a Razão Nitrogênio Ureico/Creatinina e Prognóstico de Insuficiência Cardíaca em Todo o Espectro da Fração de Ejeção

*Frequências cardíacas (FCs) ajustadas (IC 95%) de reinternação por insuficiência cardíaca (IC)/morte cardíaca/morte por todas as causas em pacientes com IC com baixa razão nitrogênio ureico/creatinina (NU/C) vs. Razão NU/C elevada.*

**Tabela 3:** – Valor preditivo de NU/C e tipo de IC para mortalidade em 2 anos

	Análise univariada		Análise multivariada	
		
	OR (CI 95%)	Valor de p	OR (IC 95%)	Valor de p
Grupo ICFEr+ NU/C baixa	1 (referência)		1 (referência)	
Grupo ICFEr+ NU/C elevada	2,201 (1,500–3,229)	0,001	1,754 (1,172–2,625)	0,006
Grupo ICFEfm+ NU/C baixa 1	1 (referência)		1 (referência)	
Grupo ICFEfm+ NU/C elevada	1,326 (0,861–2,042)	0,200	1,175 (0,744–1,853)	0,489
Grupo ICFEP+ NU/C baixa 1	1 (referência)		1 (referência)	
Grupo ICFEP+ NU/C elevada	1,874 (1,203–2,918)	0,005	1,646 (1,040–2,607)	0,033

*Ajustado para idade, sexo, etilismo, diabetes, infarto do miocárdio, arritmia, infecção pulmonar, anemia. ICFEr: insuficiência cardíaca com fração de ejeção reduzida; ICFEfm: Insuficiência cardíaca com fração de ejeção na faixa média; ICFEp: insuficiência cardíaca com fração de ejeção preservada; NU/C: nitrogênio ureico e creatinina.*

**Tabela 4:** – Análise cox de NU/C e tipo de IC para mortalidade em 2 anos

	Análise univariada		Análise multivariada	
		
	FC (IC 95%)	Valor de p	FC (IC 95%)	Valor de p
ICFEr+ NU/C baixa	1 (referência)		1 (referência)	
ICFEr+ NU/C elevada	1,376 (0,916-2,066)	0,125	1,574 (0,990-2,502)	0,055
ICFEfm+ NU/C baixa	1 (referência)		1 (referência)	
ICFEfm+ NU/C elevada	0,971 (0,625-1,510)	0,897	1,039 (0,656-1,646)	0,871
ICFEP+ NU/C baixa	1 (referência)		1 (referência)	
ICFEP+ NU/C elevada	2,543 (1,625-3,980)	<0,001	3,280 (2,002-5,375)	<0,001

*ICFEr: insuficiência cardíaca com fração de ejeção reduzida; ICFEfm: Insuficiência cardíaca com fração de ejeção na faixa média; ICFEp: insuficiência cardíaca com fração de ejeção preservada; NU/C: nitrogênio ureico e creatinina.*

### Curva ROC

**Figura 2:**
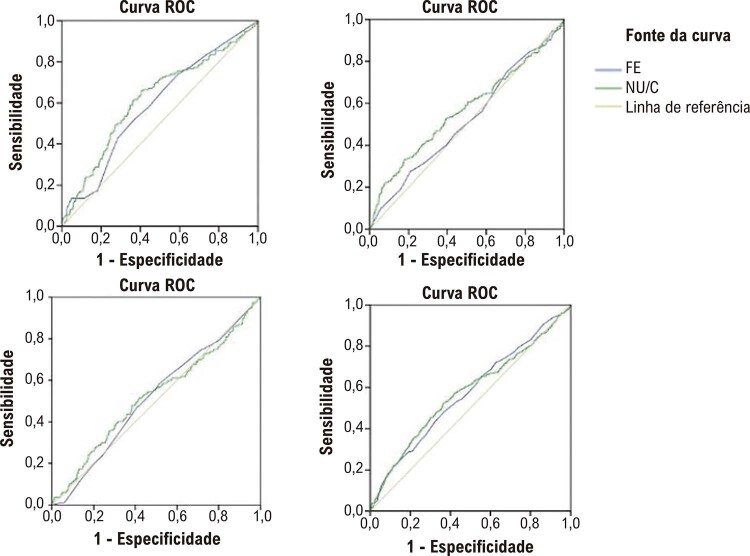
– Comparação do poder preditivo da razão NU/C e FE em pacientes com diferentes tipos de insuficiência cardíaca. FE: fração de ejeção; NU/C: nitrogênio ureico e creatinina.

A Tabela 2 mostra a linha ROC que comparou a FE com a razão NU/C na IC em todo o espectro da fração de ejeção. No grupo ICFEp e ICFEr, a área sob a curva (AUC) da razão NU/C foi maior do que a da FE, mas não foi observada em pacientes com ICFEfm. ( Figura 2 , tabelas suplementares 2 e 3). No reagrupamento de NU/C de acordo com o ponto de corte obtido da curva ROC, o risco de morte por todas as causas em dois anos no grupo razão NU/C* elevada foi maior do que no grupo razão NU/C* baixa (p<0,001), que ainda foi estatisticamente significativo após o ajuste para variáveis relevantes [FC=1,626, IC 95% (1,297–2,040), p<0,001, Tabela suplementar 4].

## Discussão

A IC é uma doença comum em humanos. O presente estudo destaca uma nova visão sobre a relação entre a razão NU/C e prognóstico clínico em pacientes com IC em todo o espectro da fração de ejeção, portanto, o prognóstico em humanos pode ser melhor avaliado. Em primeiro lugar, a razão NU/C mais elevada em pacientes com IC esteve associada a pior prognóstico; em segundo lugar, para diferentes tipos: não houve correlação entre NU/C mais elevado na ICFEr com risco de reinternação no curto e longo prazo em pacientes com IC, mas se mostrou independentemente relacionada a morte cardíaca no longo prazo e morte por todas as causas. Na ICFEfm, a razão NU/C mais elevada não teve efeito em nenhum desfecho clínico de curto ou longo prazo. Na ICFEP, embora a razão NU/C mais elevado não tenha associação com o risco de morte cardíaca, mostrou-se independentemente relacionada a reinternação de longo prazo e morte por todas as causas por IC. O motivo dessa diferença pode estar relacionado ao débito cardíaco e ao volume sanguíneo efetivo da IC em todo o espectro da fração de ejeção,^11^ mas, de acordo com as evidências existentes, não se sabe o motivo específico.

Diversos biomarcadores podem predizer o início de eventos adversos em pacientes com IC.^13^ Entre eles, o nível de NU, o nível de C e a razão NU/C são reconhecidos como indicadores clínicos da função renal atual.^14 - 17^ No entanto, NU, C são facilmente afetados por fatores não renais, sendo reabsorvidos assincronamente nos túbulos renais. Recentemente, poucos estudos relataram a liberação de arginina vasopressina (AVP) desencadeada pela relativa deficiência de volume sanguíneo em pacientes com IC. Esse fato, por sua vez, ativa o sistema neuro-hormonal, levando à vasoconstrição renal, reduzindo a filtração glomerular e a excreção de NU/C, aumentando a razão NU/C. Este histórico estabelece uma base para a razão NU/C como produtora de neuro-hormônios renais.^18^ Além disso, é importante notar que o rim aumenta diretamente a recaptação de ureia na medula renal, aumentando assim a reabsorção de sódio e água,^19^ contribuindo para um dos mecanismos fisiopatológicos do rim para A síndrome cardiorrenal na IC.^20 - 23^ Okayama et al. indicaram a razão NU/C como um indicador alternativo para fácil estimação de níveis elevados de AVP, que pode ser empregado para predizer a eficácia do tolvaptano no tratamento da IC.^24^

Okayama et al. relataram complicações em pacientes com IC concomitantemente sofrendo de insuficiência renal. Essa população de pacientes também refletiu uma razão NU/C mais elevada.^24 - 26^ Uma estreita associação da razão NU/C também foi observada com a deterioração da taxa de sobrevida.^23 , 27^ Também agrava o risco de proteinúria relacionada à IC.^23^ Estudos documentaram que a razão NU/C pode fornecer predições independentes mesmo após o ajuste do *clearance* de creatinina. Além disso, Yasumori Sujino et al. indicaram que o valor preditivo da elevação da razão NU/C na sobrevida na alta também depende da concentração sanguínea,^28^ em que uma concentração excessiva de sangue e hemodiluição tem efeito adverso na sobrevida em pacientes, sendo que isso não foi observado em pacientes com concentração sanguínea moderada e diluição da pressão arterial.^19 , 28^ Um estudo do Japão mostrou que o grupo com NU/C elevada aumenta o risco prognóstico de insuficiência cardíaca.^7^ Nossa pesquisa preenche uma lacuna inexplorada de IC em todo o espectro da fração de ejeção. Além disso, também utilizamos o ponto de corte obtido da curva ROC como base para o agrupamento e confirmamos que o grupo de NU/C elevada aumentou o risco prognóstico de insuficiência cardíaca. Shigehiko Uchino et al. afirmaram que a relação entre NU/C e mortalidade é do tipo J.^18^ Além disso, pesquisas também confirmaram que não apenas o prognóstico de pacientes com IC, mas também a razão NU/C é útil para predizer o prognóstico de outras doenças, como infarto agudo do miocárdio.^29^ A razão NU/C também pode ser explorada para predizer o prognóstico de outras doenças, como sangramento gastrointestinal em humanos,^30^ infarto agudo do miocárdio (IAM) e assim por diante.^27^ Inaguma et al. mostraram uma correlação significativa da razão NU/C mais elevada com a frequência de sintomas de IC e histórico de doença coronariana e acidente vascular cerebral isquêmico.^31^ Além disso, estudos recentes documentaram que níveis elevados de NU, NU/C são preditores independentes de gravidade e sobrevida na COVID-19.^32^

Nosso estudo fornece uma base para o manejo eficaz de pacientes com IC e um novo índice para o prognóstico de humanos. Atualmente, há uma grande quantidade de evidências de que está associada ao aumento do risco de IC, mas há escassez de evidências sobre a relação entre NU/C e o prognóstico da IC em todo o espectro da fração de ejeção. O presente estudo analisou a relação entre NU/C e o prognóstico de curto ou longo prazo de pacientes com IC em todo o espectro da fração de ejeção.

### Limitações

O presente estudo apresenta diversas limitações. Em primeiro lugar, não foram levados em consideração outros fatores predisponentes que podem afetar a razão NU/C, incluindo o uso de drogas como corticosteroides e alguns antibióticos. Em segundo lugar, porque este estudo é um estudo observacional, outros fatores de confusão que afetam os resultados não podem ser excluídos, mesmo após análise ajustada. Por fim, mais estudos são necessários para esclarecer melhor o papel da NU/C na IC em todo o espectro da fração de ejeção. Apesar dessas limitações, nosso estudo enfatizou que os pacientes do grupo NU/C elevada tiveram mau prognóstico no longo prazo, e não houve correlação significativa entre NU/C elevada com prognóstico em pacientes com ICFEfm.

## Conclusão

O grupo NU/C elevada está associado ao risco de mau prognóstico da ICFEP, não sendo inferior ao valor preditivo da FEVE.

## *Material suplementar

Para informação adicional, por favor,clique aqui


