# Supplementary material for: Relação entre a Razão Nitrogênio Ureico/Creatinina e Prognóstico de Insuficiência Cardíaca em Todo o Espectro da Fração de Ejeção
Source: Arq Bras Cardiol. 2023 Mar 20;120(3):e20220427. [Article in Portuguese] doi: 10.36660/abc.20220427 (PMC10392858; doi:10.36660/abc.20220427)
Supplement: Supplementary file 1 [file 2022-0427_AO_materialsuplementar.pdf]

Supplementary table 1 - Baseline characteristics of different ejection fraction group with symptomatic HF

Clinical characteristic	Total	HfrEF Low BUN/Cr*	HfrEF High BUN/Cr*	*P value*	HfmrEF Low BUN/Cr*	HfmrEF High BUN/Cr*	*P value*	HfpEF Low BUN/Cr*	HfpEF High BUN/Cr*	*P value*
Age	70(61-79)	67.5(61-78)	75(64-82)	0.162	76.5(65.25-84)	77(64-82)	0.560	77(66-83)	77(67-83)	0.897
Female	794(37.8)	86(32.5)	130(53.1)	＜0.001	128(30.9)	122(42.2)	0.001	68(30)	89(39.9)	0.017
Smoke	288(13.8)	106(40.5)	99(40.4)	0.532	175(42.4)	115(39.9)	0.285	79(34.8)	62(28.1)	0.075
Drink	849(40.6)	43(16.5)	38(15.5)	0.424	62(15)	30(10.5)	0.048	25(11)	19(8.6)	0.247
Comorbidities										
hypertention	1235(58.8)	187(70.6)	154(62.9)	0.040	239(57.7)	166(57.4)	0.500	119(52.4)	109(48.9)	0.255
Af	578(27.5)	54(20.4)	58(23.7)	0.214	110(26.6)	105(36.3)	0.004	80(35.2)	92(41.3)	0.112
Diabetes	646(30.8)	99(37.4)	110(44.9)	0.051	112(27.1)	85(29.4)	0.274	50(22)	59(26.5)	0.162
AMI	951(45.3)	129(48.7)	108(44.1)	0.171	200(48.3)	120(41.5)	0.044	92(40.5)	64(28.7)	0.006
CAD	1668(79.5)	197(78.5)	209(51.5)	0.305	339(83.7)	223(39.7)	0.003	179(80.6)	156(68.4)	0.002
Arrhythmia	968(46.1)	120(45.3)	107(43.7)	0.391	190(45.9)	160(55.4)	0.008	104(45.8)	119(53.4)	0.066
Renal insufficient	423(20.2)	121(45.7)	60(24.5)	＜0.001	79(19.1)	52(18.0)	0.396	46(20.3)	48(21.5)	0.416
Pulmonary infection	575(27.4)	69(26)	65(26.5)	0.490	114(27.5)	100(34.6)	0.028	75(33)	80(35.9)	0.297
Anemia	310(14.8)	37(14.0)	48(19.6)	0.056	62(15)	38(13.1)	0.285	43(18.9)	50(22.4)	0.213
Laboratory measurements										
NT-ProBNP(pg/mL)	6300.1±7467.7	7423.8±8085.0	7585.8±8451.8	0.839	5818.0±6894.7	8219.6±8778.3	＜0.001	6111.6±6751.1	8149.5±8821.8	0.022
Lactate dehydrogenase(U/L)	331.0±344.1	351.6±402.4	330.2±324.8	0.519	325.8±283.0	346.3±357.9	0.407	312.3±445.7	334.6±379.65	0.577
Aspertate aminotransferase(U/L)	19(14-29)	19(14-29)	22(15-33.50)	0.105	20(14-31.9)	22(16-33.3)	0.041	22(15.3-32)	23(17-35.6)	0.229
S-Creatinine (mg/dL)	105.4±64.3	165.48±99.9	101.3±40.5	＜0.001	111.3±63.1	92.3±39.8	＜0.001	112.1±74.9	95.1±54.2	0.006
Urea nitrogen(mmol/L)	9.5±23.8	10.2±4.5	13.4±19.7	＜0.001	6.5±3.2	12.0±28.5	＜0.001	6.7±4.0	17.6±59.5	＜0.001
Hemoglobin(g/L)	127(111-142)	119.5(95.3-144)	122(102-136)	0.365	123.5(106-137.8)	121.5(105-141)	0.567	123.5(104.3-137)	115(100.5-132.8)	0.229
Red Blood cells(*10^{12}/L)		4.3±0.9	4.3±0.9	0.708	4.3±0.7	4.4±1.0	0.225	4.1±0.7	4.0±0.8	0.167

Druge history									
ACEI	709(33.8)	89(33.6)	82(33.5)	0.527	134(32.4)	86(29.8)	0.258	65(28.6)	53(23.8)	0.143
ARB	511(24.3)	73(27.5)	63(25.7)	0.357	109(26.3)	64(22.1)	0.119	50(22)	43(19.3)	0.274
β-blocker	1323(63.0)	183(69.1)	145(59.2)	0.013	263(63.5)	170(58.8)	0.119	115(50.7)	117(52.5)	0.386
diuretic	1367(65.1)	198(74.7)	178(72.7)	0.334	263(63.5)	205(70.9)	0.024	141(62.1)	146(65.5)	0.260
digitalis	575(27.4)	72(27.2)	74(30.2)	0.255	90(21.7)	92(31.8)	0.002	72(31.7)	76(34.1)	0.333
Cardiac color Doppler ultrasound									
LA(mm)	42.6±6.4	42.6±6.4	42.2±7.5	0.481	42.5±7.1	43.6±7.5	0.056	43.8±8.3	42.9±9.7	0.882
LV(mm)	56.4±10.3	58.2±10.1	56.5±10.6	0.068	56.0±10.0	57.4±10.3	0.076	55.7±10.7	55.3±11.2	0.677
RA(mm)	39.3±8.8	37.8±6.7	39.1±8.5	0.212	38.6±8.3	41.0±9.2	0.019	40.2±8.3	42.2±11.5	0.129
RV(mm)	24(18-30)	26(19.3-31)	25(18-30)	0.276	24(18-30)	25(18.8-31.3)	0.639	30(24-33)	28(21-34)	0.391

Supplementary Table 2：Comparison of predictive power of BUN/Cr and EF in different types of heart failure patients Comparison of the areas under the ROC curve.（total HF）

		AUC	AUC(95%CI)	P
HFpEF	EF	0.588	0.533-0.644	0.002
	BUN/Cr	0.617	0.516-0.674	＜0.001
HFmrEF	EF	0.516	0.466-0.565	0.537
	BUN/Cr	0.523	0.471-0.575	0.　371
HFrEF	EF	0.518	0.471-0.574	0.441
	BUN/Cr	0.568	0.519-0.616	0.004

Supplementary Table 3: Unadjusted HRs (95%CI) of HF re-hospitalization/cardiac death/all-cause death.

	HFrEF		HFmrEF		HFpEF	
	OR（95%CI）	*P* value	OR(95%CI)	*P* value	OR（95%CI）	*P* value
Rehospitalization for HF						
3 months	1.943 (0.923-4.088)	0.080	1.477 (0.680-3.213)	0.325	1.962 (0.897-4.292)	0.092
12 months	1.010 (0.641-1.591)	0.966	0.950 (0.587-1.538)	0.835	1.818 (1.114-2.966)	0.017
24 months	0.874 (0.563-1.356)	0.547	0.988 (0.629-1.553)	0.959	1.791 (1.118-2.869)	0.015
Cardiac death						
3 months	2.897 (1.319-6.360)	0.008	0.528(0.157-1.776)	0.302	1.134 (0.463-2.773)	0.783
12 months	1.921 (1.155-3.195）	0.012	0.742(0.398-1.383)	0.348	1.283 (0.717-2.296)	0.401
24 months	2.018 (1.334-3.052)	0.001	0.965(0.591-1.574)	0.886	1.187 (0.721-1.956)	0.500
All-cause death						
3 months	2.901 (1.449-5.809)	0.003	0.858(0.323-2.280)	0.759	1.511 (0.712-3.209)	0.282
12 months	2.158 (1.370-3.399	0.001	1.053(0.620-1.788)	0.848	1.922 (1.169-3.161)	0.010
24 months	2.201 (1.500-3.229)	＜0.001	1.326(0.861-2.042)	0.200	1.874 (1.203-2.918)	0.005

Supplementary table 4 - The cox analysis of BUN/Cr and HF for 2-year mortality

	Univariate analysis		Multivariate analysis	
	HR (95%CI)	*P* value	HR (95%CI)	*P* value
Low BUN/Cr	1 (reference)		1 (reference)	
High BUN/Cr	1.336(1.047-1.706)	0.023	1.323(1.021-1.714)	0.034
Low BUN/Cr*	1 (reference)		1 (reference)	
High BUN/Cr*	1.499(1.213-1.853)	＜0.001	1.626(1.297-2.040)	＜0.001

BUN/Cr*：Adjust for female，Renal insufficient，Cr，BUN
