# Supplementary material for: Relação entre a Razão Nitrogênio Ureico/Creatinina e Prognóstico de Insuficiência Cardíaca em Todo o Espectro da Fração de Ejeção
Source: Arq Bras Cardiol. 2023 Mar 20;120(3):e20220427. [Article in Portuguese] doi: 10.36660/abc.20220427 (PMC10392858; doi:10.36660/abc.20220427)
Supplement: Translation

# Translation: Relationship between BUN/Cr and Prognosis of HF Across the Full Spectrum of Ejection Fraction

**Authors:** Yuan Kang, Conglin Wang, Xiaojing Niu, Zhijing Shi, Mingxue Li, Jianli Tian

**Affiliations:** 1 China Department of Geriatrics, Tianjin Medical University General Hospital, Tianjin – China

**Keywords:** Heart Failure, BUN/Cr, Ejection fraction, Prognosis

## Abstract

**Background:**

In patients with heart failure (HF), due to the relative deficiency of blood volume, neurohormone system activation leads to renal vasoconstriction, which affects the content of blood urea nitrogen (BUN) and creatinine (Cr) in the body, while BUN and Cr are easily affected by other factors. Therefore, BUN/Cr can be used as another marker for the prognosis of HF.

**Objective:**

Explore the prognosis of adverse outcome of HF in the high BUN/Cr group compared with the low BUN/Cr group across the full spectrum of ejection fraction.

**Methods:**

From 2014 to 2016, symptomatic hospitalized HF patients were recruited and followed up to observe adverse cardiovascular outcomes. Logistic analysis and COX analysis were performed to determine significance. p-values <0.05 were considered statistically significant.

**Results:**

In the univariate logistic regression analysis, the high BUN/Cr group had a higher risk of adverse outcome in heart failure with reduced ejection fraction (HFrEF) and heart failure with preserved ejection fraction (HFpEF). Multivariate logistic regression analysis showed that the risk of cardiac death in the HFrEF group was higher than that in the low BUN/Cr group, while the risk of all-cause death was significant only in 3 months (p<0.05) (Central Illustration). The risk of all-cause death in the high BUN/Cr in the HFpEF group was significantly higher than that in the low BUN/Cr group at two years.

**Conclusion:**

The high BUN/Cr group is related to the risk of poor prognosis of HFpEF, and is not lower than the predictive value of left ventricular ejection fraction (LVEF).

## Introduction

In recent times, heart failure (HF) has been often found in the geriatric population. According to the 2021 guidelines of the European Heart Association, patients with HF are categorized into (1) Heart failure with reduced ejection fraction (HFrEF), where reduced left ventricular ejection fraction (LVEF) is defined as ≤40%; (2) Heart failure with mid-range ejection fraction (HFmrEF), including patients with LVEF between 41% and 49%; and (3) Heart failure with preserved ejection fraction (HFpEF), where patients present LVEF ≥49%.^1^Although novel strategies are being continuously introduced to combat HF, it persists as one of the problems with the highest mortality and readmission rates among inpatients. Some studies have documented the association of the mortality of patients with HF with the lack of effective blood volume,^2^but other studies have suggested that patients with HF would have excessive activation of different neurohormones, such as renin–angiotensin–aldosterone system (RAAS) and sympathetic nervous system (SNS),^3^resulting in venous congestion and renal insufficiency. The above mechanisms will have a certain impact on the prognosis.

In patients with HF, renal insufficiency is also attributed to the decrease of myocardial contractility.^4^Apart from decreasing estimated glomerular filtration rate (eGFR),^5^renin also increases the reabsorption of water and salt, leading to enhanced blood urea nitrogen (BUN).^6^Hence, serum creatinine (Cr) and BUN are considered effective clinical indicators of poor prognosis. Under physiological conditions, BUN can be filtered freely in the glomerulus, but 30% to 40% is reabsorbed in the renal tubule.^5 , 7^BUN reabsorption also increases owing to excessive activation of neurohormones in patients with HF,^8^while protein intake, increased catabolism, and other factors also alter BUN levels.^9^Cr can be filtered freely in the glomerulus, though it is not reabsorbed in the renal tubule. Cr is also easily affected by diet, and other factors.^10 , 11^Therefore, BUN/Cr ratio may be an indicator of renal dysfunction and a measure of neurohormone and sympathetic nerve activity. Not only that, the ratio is also related to adverse events in patients with HF.^6^

Although the normal BUN/Cr ratio is unknown, previous studies revealed that BUN/Cr >25.5 is an independent risk factor for predicting death in patients with acute or chronic HF.^7^BUN is seen as a reflection and decrease in cardiac output. BUN is proportional to the hemodynamic status of the damaged prognostic marker.^9^It is known that cardiac output is diverse in patients with HF across the full spectrum of ejection fraction. However, there is no description of the predictive ability of BUN/Cr in patients with HF across the full spectrum of ejection fraction. Therefore, the present study compares the prognosis of HF across the full spectrum of ejection fraction by BUN/Cr at admission.

## Methods

### Study Population

This retrospective observational study was conducted on 2,255 symptomatic HF patients who visited the outpatient departments of Tianjin Medical University General Hospital and Tianjin Thoracic Hospital from February 2014 to June 2016 in Tianjin, China. Inclusion criteria: (1) Patients ≥18 years of age with symptomatic HF (NYHA functional class III–IV); (2) BUN, Cr, and other laboratory indexes estimated in the first 24 hours of admission. Exclusion criteria: (1) Patients with incomplete indicators; (2) Patients without prognostic information; (3) Patients with severe tuberculosis or malignant tumor. HF was diagnosed following the 2021 European Society of Cardiology (ESC) guidelines and was examined by at least two doctors with attendance rates, based on the patient’s symptoms, signs, laboratory results, and cardiac function assessment. All patients signed an informed consent form to participate in the study. The research program is in line with the principles of the Helsinki Declaration and approved by the Medical Ethics Committee of the General Hospital of Tianjin Medical University (Ethics Committee approval No.: IRB2017.029–01, Registration No.: CHICCTR-ERC-17011820).

### Baseline Information and Laboratory Tests

For all subjects, general demographic information, comorbidities, medication, echocardiography data, and laboratory results were recorded. Diagnosis of HF and grouping according to ejection fraction was based on the 2016 European Society of Cardiology (ESC) guidelines.^12^Since most of the patients were not admitted on an empty stomach, peripheral venous blood was collected on the second day of admission, and the laboratory indexes were estimated. All patients underwent transthoracic color Doppler echocardiography within 48 hours of admission, the instruments used are internationally recognized standard clinical equipment, and the ultrasound results were evaluated by professionally trained clinicians.

### Study Population: Follow-up and Study Endpoints

The endpoints of this study were defined as readmission for HF, cardiac death, and all-cause death, and the tenure for the clinical result was 3 months, 12 months, and 24 months, respectively. This study was followed up for 2 years through outpatient clinic visits or telephone communication. The follow-up staffs were all trained clinicians. Data collection and follow-up of all patients enrolled in the group were conducted by the clinicians on paper questionnaires. The paper version of the medical records was stored. Upon completion of follow-up, two special clinicians selected 10% of the cases from the patients in the group for data check, including checks of paper questionnaires and telephone follow-up. Of the 2,099 patients, all patients completed a two-year follow-up without any intervention, and the researchers obtained prognostic information.

### Statistical analysis

According to different ejection fractions, 2,099 subjects were divided into three groups, and according to the level of BUN/Cr, the subjects were divided into two subgroups (BUN/Cr≤25.5 and BUN/Cr≤25.5). Firstly, we used the Kolmogorov-Smirnov test to check normality. Independent T-test or Wilcoxon Rank-Sum test was used for continuous variables, in which variables satisfying normal distribution were represented by mean±standard deviation, whereas median and quartile spacing was used to represent variables with non-normal distribution. Categorical variables are expressed as absolute numbers and percentages. Pearson’s chi-square test or Fisher’s exact test was adopted to compare the constituent ratios among groups, and the baseline characteristics of the subjects were obtained. Thereafter, logistic regression analysis was employed to compare the prognosis of 3 months, 12 months, and 24 months in different BUN/Cr groups. The significant demographic variables and comorbidities found in HF across the full spectrum of ejection fraction in univariate analysis were used to adjust confounding factors, including age, sex, alcohol consumption, diabetes, myocardial infarction, arrhythmia, pulmonary infection, anemia, etc. The results were expressed by odds ratio (OR) and 95% confidence interval (CI). According to the different groups of ejection fraction, we then adjusted the common significant demographic factors and the confounding factors of laboratory indicators, such as sex, age, BUN, Cr, hemoglobin, and so on. Taking the group of low BUN/Cr (BUN/Cr>25.5) as a reference, logistic regression was employed to analyze the two-year all-cause mortality. ROC curve analysis helped to evaluate the BUN/Cr group prediction of two-year all-cause mortality. In addition, according to the cut-off point of ROC curve (cut-off point=20.4043), BUN/Cr was divided into a new group and the related baseline characteristics and prognostic factors were compared (High BUN/Cr*≤20.4043, Low BUN/Cr*>20.4043). All measurements were bilateral, and p<0.05 was considered statistically significant. All statistical analyses were carried out using SPSS statistical software (version 22.0) IBM Corp.

## Result

### Clinical characteristics

A total of 124 patients with no BUN or Cr and 31 patients with severe tuberculosis or malignant tumors were excluded, and the remaining 2,099 symptomatic HF patients were enrolled in this study. The average age of the 2,099 patients included in this study was 70 (61-79), of which 794 were women (37.8%). The mean Cr in this population was 105.4±64.3 mg/dL, and the mean BUN was 9.5±23.8 mmol/L ( Table 1 , supplementary table 1).

**Table 1:** – Baseline characteristics of different ejection fraction groups with symptomatic HF

Clinical characteristics	Total	HFrEF		p value	HFmrEF		p value	HFpEF		p value
		
		Low BUN/Cr	High BUN/Cr		Low BUN/Cr	High BUN/Cr		Low BUN/Cr	High BUN/Cr	
Age	70 (61–79)	66 (58–76)	70 (61–79)	0.004	72 (62–80)	74 (63–80)	0.369	74 (64–81)	72 (63–82)	0.820
Female	794 (37.8)	299 (37.6)	88 (58.3)	<0.001	193 (33.6)	57 (44.5)	0.013	109 (32.3)	48 (43.2)	0.023
Smoke	288 (13.8)	132 (16.8)	20 (13.2)	0.168	237 (41.3)	53 (41.7)	0.502	115 (34.1)	26 (23.4)	0.022
Drink	849 (40.6)	368 (46.5)	50 (33.1)	0.001	79 (13.8)	13 (10.2)	0.176	38 (11.3)	6 (5.4)	0.046
**Comorbidities**										
Hypertension	1235 (58.8)	507 (63.8)	95 (62.9)	0.454	332 (57.7)	73 (57.0)	0.480	184 (54.3)	44 (39.6)	0.005
AF	578 (27.5)	152 (19.1)	39 (25.8)	0.041	161 (28.0)	54 (42.2)	0.001	122 (36.0)	50 (45)	0.056
Diabetes	646 (30.8)	266 (33.5)	74 (49.0)	<0.001	161 (28.0)	36 (28.1)	0.528	76 (22.4)	33 (29.7)	0.077
AMI	951 (45.3)	412 (51.8)	63 (41.7)	0.014	276 (48.5)	44 (34.4)	0.003	128 (37.8)	28 (25.2)	0.010
CAD	1668 (79.5)	648 (81.5)	123 (81.5)	0.533	467 (81.2)	95 (74.2)	0.050	256 (75.5)	79 (71.2)	0.215
Arrhythmia	968 (46.1)	319 (40.1)	76 (50.3)	0.013	275 (47.8)	75 (58.6)	0.017	161 (47.5)	62 (55.9)	0.078
Renal insufficiency	423 (20.2)	160 (20.1)	38 (25.2)	0.101	102 (17.7)	29 (22.7)	0.123	68 (20.1)	26 (23.4)	0.264
Pulmonary infection	575 (27.4)	163 (20.5)	43 (28.5)	0.021	161 (28.0)	53 (51.4)	0.002	114 (33.6)	41 (36.9)	0.299
Anemia	310 (14.8)	86 (10.8)	31 (20.5)	0.001	75 (13.0)	25 (19.5)	0.042	66 (19.5)	27 (24.3)	0.168
**Laboratory measurements**										
NT-ProBNP (pg/mL)	6300.1± 7467.7	5219.2± 6419.3	7931.8± 9352.9	<0.001	6352.7± 7504.5	8990.8± 8875.4	0.002	8990.8± 8875.4	8990.8± 8875.4	0.501
Lactate dehydrogenase (U/L)	331.0± 344.1	336.6± 12.3	309.8± 247.3	0.035	329.0± 288.3	357.2± 416.5	0.183	340.0± 468.3	271.8± 146.37	0.011
Aspartate aminotransferase (U/L)	23.2 (16.5–43.2)	22 (16–45.9)	23.5 (16–44.5)	0.942	24 (17–45.7)	25 (16.8–44.6)	0.820	18 (12–33.1)	17 (11.7–38.3)	0.780
S-Creatinine (mg/dL)	105.4±64.3	111.7±72.9	86.7±41.5	0.004	107±58.1	87.6±18.3	<0.001	109.3±69.7	86.7±49.53	0.046
Urea nitrogen (mmol/L)	9.5±23.8	7.3±4.0	15.4±25.5	<0.001	7.3±3.7	16.1±43	<0.001	7.7±4.7	26.2±84.4	<0.001
Uric acid (umol/L)	386.5± 150.4	386.5± 133.5	432.9± 177.3	<0.001	367.4± 142.1	441.9± 192.8	<0.001	372.5± 148.1	402.2± 187.9	0.038
Hemoglobin (g/L)	127 (111–142)	131 (116–146)	126 (108.3–141)	0.034	127 (112.2–142)	122.5 (105–141)	0.439	123 (106–137)	118 (105.5–135.5)	0.455
Red blood cell distribution	13 (12.3–14.1)	12.9 (12.1–13.7)	13.2 (12.4–14.7)	<0.001	13 (12.2–14.1)	13.4 (12.7–15)	0.002	13.2 (12.3–14.3)	14 (13–15.2)	<0.001
Red blood cells (*1012/L)		4.4±0.8	4.3±0.9	0.153	4.3±0.7	4.4±1.2	<0.001	4.1±0.7	3.9±0.8	0.586
Red blood cell specific volume	55.9±25.5	53.4±23.2	57.1±26.5	<0.001	53.6±24.2	55.3±27.1	0.034	61.5±27.5	64.9±29.9	0.006
**Drug history**										
ACEI	709 (33.8)	320 (40.3)	51 (33.8)	0.080	189 (32.9)	31(24.2)	0.034	94 (27.7)	24 (21.6)	0.125
ARB	511 (24.3)	213 (26.8)	32 (21.2)	0.089	141 (24.5)	32(25.0)	0.495	74 (21.8)	19 (17.1)	0.177
β-blocker	1323 (63.0)	567 (71.3)	91 (60.3)	0.005	363 (63.1)	70(54.7)	0.048	172 (50.7)	60 (54.1)	0.310
Diuretics	1367 (65.1)	503 (63.3)	109 (72.2)	0.021	381 (66.3)	87(68.0)	0.397	216 (63.7)	71 (64.0)	0.529
Digitalis	575 (27.4)	192 (24.2)	53 (35.1)	0.004	143 (24.9)	39(30.5)	0.117	107 (31.6)	41 (36.9)	0.176
**Cardiac color Doppler ultrasound**										
LA (mm)	42.5±7.6	41.9±6.9	41.6±7.3	0.915	42.9±7.1	43.4±7.8	0.141	42.9±8.3	42.8±10.9	0.002
LV (mm)	56.4±10.3	56.8±10.1	56.2±10.1	0.116	56.6±9.9	56.3±11.3	0.020	56.0±10.5	53.9±12.1	0.090
RA (mm)	39.3±8.8	37.1±6.8	40.2±8.4	0.038	39.2±8.3	56.3±11.3	0.056	41.1±9.9	41.9±11.3	0.109
RV (mm)	24 (18–30)	23 (16.5–32)	23 (18.1–31.2)	0.723	22 (18.2–30.6)	23 (17.4–31.9)	0.686	28 (20.2–32.5)	28 (20.3–34.5)	0.698

*HFrEF: heart failure with reduced ejection fraction; HFmrEF: heart failure with mid-range ejection fraction; HFpEF: heart failure with preserved ejection fraction; BUN/Cr: blood urea nitrogen and creatinine; AF: atrial fibrillation; AMI: acute myocardial infarction; CAD: coronary artery disease; ACEI: angiotensin-converting enzyme inhibitors; ARB: angiotensin receptor blockers; LA: left atrium; LV: left ventricle; RA: right atrium; RV: right ventricle.*

### Survival analysis

In the logistic regression analysis of the HFrEF group with unadjusted variables, compared with the low BUN/Cr group, the risk of cardiac death in the high BUN/Cr group was higher than that in the low BUN/Cr group at 3 months, 12 months and 24 months, and the risk of all-cause death in 3 months was 2.062 times higher than that in the low BUN/Cr group ( Table 2 ). In the HFmrEF group, there was no significant difference in clinical outcome at each follow-up point. In the HFpEF group, the risk of rehospitalization due to HF in the high BUN/Cr group was higher than that in the low BUN/Cr group at the 12^th^and 24^th^months of follow-up, and the risk of all-cause death was 2.1 times higher than that in the low BUN/Cr group (p<0.005). Through logistics regression analysis, we found that after adjusting the corresponding confounding factors, the risk of cardiac death and 3-month all-cause death at each follow-up point in the HFrEF group was still higher than that in the low BUN/Cr group, and the risk of HF rehospitalization and all-cause death in the HFpEF group was still significantly higher than that in the low BUN/Cr group at 12 and 24 months ( Central Illustration , supplementary table 2). For the all population with HF, the risk of all-cause death in the high BUN/Cr group was significantly higher than that in the low BUN/Cr group in the HFpEF group. In the HFrEF and HFpEF group, compared with the low BUN/Cr group, the high BUN/Cr group had a significantly higher 2-year all-cause death rate after adjusting for mixed factors ( Table 3 ). In the HFpEF group, this significant trend was also observed in the Cox analysis, which the risk of the high BUN/Cr group was 3.280 times higher than that of the low BUN/Cr group after adjusting for related risks (p<0.001, Table 4 ). Kaplan–Meier survival curves in HFpEF with BUN/Cr ratios were shown in figure 1 (p<0.001).

**Table 2:** – Adjusted HRs (95%CI) of HF re-hospitalization/cardiac death/all-cause death in HF patients with low BUN/Cr vs. high BUN/Cr

Clinical prognosis	HFrEF	p value	HFmrEF	p value	HFpEF	p value
		
	Adjusted OR (95 %CI)		Adjusted OR (95 %CI)		Adjusted OR (95 %CI)	
**Rehospitalization for HF**						
3 months	2.234 (0.932–5.351)	0.071	1.346 (0.519–3.493)	0.541	2.058 (0.872–4.855)	0.099
12 months	0.790 (0.465–1.343)	0.384	0.892 (0.529–1.506)	0.67	1.849 (1.103–3.101)	0.02
24 months	0.711 (0.429–1.177)	0.185	0.948 (0.582–1.544)	0.829	1.784 (1.087–2.929)	0.022
**Cardiac death**						
3 months	2.508 (1.075–5.850)	0.033	0.456 (0.128–1.624)	0.226	1.091 (0.426–2.794)	0.855
12 months	1.972 (1.118–3.480)	0.019	0.766 (0.397–1.480)	0.427	1.225 (0.656–2.288)	0.524
24 months	2.062 (1.287–3.301)	0.003	1.046 (0.623–1.758)	0.864	1.095 (0.644–1.860)	0.738
**All-cause death**						
3 months	2.608 (1.221–5.572)	0.013	0.748 (0.269–2.075)	0.577	1.717 (0.768–3.838)	0.188
12 months	1.543 (0.863–2.761)	0.144	1.098 (0.623–1.935)	0.747	2.101 (1.227–3.598)	0.007

*Adjusted for sex, age, BUN, Cr, hemoglobin. HFrEF: heart failure with reduced ejection fraction; HFmrEF: heart failure with mid-range ejection fraction; HFpEF: heart failure with preserved ejection fraction.*

**Central Illustration:** : Relationship between BUN/Cr and Prognosis of HF Across the Full Spectrum of Ejection Fraction

*Adjusted heart rates (HRs) (95%CI) of heart failure (HF) re-hospitalization/cardiac death/all-cause death in HF patients with low BUN/Cr vs. high BUN/Cr.*

**Table 3:** – Predictive value of BUN/Cr and HF type for 2-year mortality

	Univariate analysis		Multivariate analysis	
		
	OR (95%CI)	p value	OR (95%CI)	p value
HFrEF+Low BUN/Cr group	1 (reference)		1 (reference)	
HFrEF+High BUN/Cr group	2.201 (1.500–3.229)	0.001	1.754 (1.172–2.625)	0.006
HFmrEF+Low BUN/Cr group	1 (reference)		1 (reference)	
HFmrEF+High BUN/Cr group	1.326 (0.861–2.042)	0.200	1.175 (0.744–1.853)	0.489
HFpEF+Low BUN/Cr group	1 (reference)		1 (reference)	
HFpEF+High BUN/Cr group	1.874 (1.203–2.918)	0.005	1.646 (1.040–2.607)	0.033

*Adjusted for age, sex, alcohol consumption, diabetes, myocardial infarction, arrhythmia, pulmonary infection, anemia. HFrEF: heart failure with reduced ejection fraction; HFmrEF: heart failure with mid-range ejection fraction; HFpEF: heart failure with preserved ejection fraction; BUN/Cr: blood urea nitrogen and creatinine.*

**Table 4:** – The cox analysis of BUN/Cr and HF type for 2-year mortality

	Univariate analysis		Multivariate analysis	
		
	HR (95%CI)	p value	HR (95%CI)	p value
HFrEF+Low BUN/Cr	1 (reference)		1 (reference)	
HFrEF+High BUN/Cr	1.376 (0.916-2.066)	0.125	1.574 (0.990-2.502)	0.055
HFmrEF+Low BUN/Cr	1 (reference)		1 (reference)	
HFmrEF+High BUN/Cr	0.971 (0.625-1.510)	0.897	1.039 (0.656-1.646)	0.871
HFpEF+Low BUN/Cr	1 (reference)		1 (reference)	
HFpEF+High BUN/Cr	2.543 (1.625-3.980)	<0.001	3.280 (2.002-5.375)	<0.001

*Adjusted for female. HFrEF: heart failure with reduced ejection fraction; HFmrEF: heart failure with mid-range ejection fraction; HFpEF: heart failure with preserved ejection fraction; BUN/Cr: blood urea nitrogen and creatinine.*

**Figure 1:** – Kaplan–Meier survival curves in patients with high and low BUN/Cr ratios. BUN/Cr: blood urea nitrogen and creatinine.

### ROC curve

Table 2 shows the ROC line which compared EF and BUN/Cr in HF across the full spectrum of ejection fraction. In the group of HFpEF and HFrEF, the AUC of BUN/Cr was larger than that of EF, but it was not observed in patients with HFmrEF ( Figure 2 , tables 2 and 3 ). In the regrouping of BUN/Cr according to the cut-off point obtained from the ROC curve, the risk of 2-year all-cause death in the high BUN/Cr* group was higher than that in the low BUN/Cr* group (p<0.001), which was still statistically significant after adjusting for relevant variables [HR=1.626, 95%CI (1.297–2.040), p<0.001, supplementary table 4].

**Figure 2:** – Comparison of predictive power of BUN/Cr and EF in patients with different types of heart failure. EF: ejection fraction; BUN/Cr: blood urea nitrogen and creatinine.

## Discussion

HF is a common disease in humans. The present study highlights a new insight into the relationship between BUN/Cr and clinical prognosis in patients with HF across the full spectrum of ejection fraction, thus prognosis in humans can be better judged. Firstly, higher BUN/Cr in patients with HF was associated with poor prognosis; secondly, for different types: there was no correlation between higher BUN/Cr in HFrEF with short-term and long-term risk of rehospitalization in patients suffering from HF, but it was independently related to long-term cardiac death and all-cause death. In HFmrEF, the higher BUN/Cr exerted no effect on any short-term or long-term clinical results. In HFpEF, though higher BUN/Cr had no association with the risk of cardiac death, it was independently related to long-term rehospitalization and all-cause death due to HF. The reason for this difference may be related to the cardiac output and effective blood volume of HF across the full spectrum of ejection fraction,^11^but according to the existing evidence, the specific reason is not known.

Several biomarkers can predict the onset of adverse events in patients with HF.^12^Among them, BUN level, Cr level, and BUN/Cr ratio are recognized as clinical indicators of renal function at present.^13 - 17^Nonetheless, BUN, Cr is easily affected by non-renal factors and is reabsorbed asynchronously in the renal tubules. Recently, few studies have reported the release of arginine vasopressin (AVP) triggered by the relative deficiency of blood volume in patients with HF. This, in turn, activates the neurohormone system, leading to renal vasoconstriction, reducing glomerular filtration and BUN/Cr excretion, ultimately increasing the BUN/Cr ratio. This background lays a foundation for BUN/Cr as a producer of renal neurohormones.^18^Furthermore, it is worth noting that the kidney directly enhances the reuptake of urea in the renal medulla, thereby escalating the reabsorption of sodium and water.^19^This contributes to one of the pathophysiological mechanisms of kidney for HF-cardiorenal syndrome.^20 - 23^Okayama et al. claimed BUN/Cr as an alternative indicator for easy estimation of elevated AVP levels, which can be employed to predict the efficacy of tolvaptan in the treatment of HF.^24^

Okayama et al. reported complications in patients with HF concurrently suffering from renal insufficiency. This patient population also reflected a higher BUN/Cr.^24 - 26^A close association of BUN/Cr was also observed with the deterioration of survival rate.^23 , 27^It also aggravates the risk of proteinuria related to HF.^23^Studies have documented that BUN/Cr can provide independent predictions even after adjusting creatinine clearance. Moreover, Yasumori Sujino et al. reported that the predictive value of high BUN/Creatinine on survival at discharge also relies on blood concentration,^28^whereby an excessive blood concentration and hemodilution has an adverse effect on survival in patients, while it was not observed in patients with moderate blood concentration and blood pressure dilution.^19 , 28^A study from Japan showed that the high BUN/Cr group increases the prognostic risk of heart failure.^7^Our research fills an unexplored gap of HF across the full spectrum of ejection fraction. In addition, we also used the cut-off point obtained from the ROC curve as the basis for grouping, and confirmed that the high BUN/Cr group increased the prognostic risk of heart failure. Shigehiko Uchino et al. claimed that the relationship between BUN/Cr and mortality is J-type.^18^Furthermore, research also confirmed that not only the prognosis of patients with HF, but also BUN/Cr is useful to predict the prognosis of other diseases such as acute myocardial infarction.^29^BUN/Cr can also be exploited to predict the prognosis of other diseases, such as gastrointestinal bleeding in humans,^30^acute myocardial infarction (AMI), and so on.^27^Inaguma et al. revealed a significant correlation of the higher BUN/Cr ratio with the frequency of HF symptoms and the history of coronary heart disease and ischemic stroke.^31^Moreover, recent studies have documented that elevated levels of BUN, BUN/Cr are independent predictors of COVID-19 severity and survival.^32^

Our study provides a basis for the effective management of patients with HF, and provides a new index for the prognosis of humans. At present, there is a large amount of evidence that it is associated with the increased risk of HF, but there is a dearth of evidence on the relationship between BUN/Cr and the prognosis of HF across the full spectrum of ejection fraction. The present study analyzed the relationship between BUN/Cr and short-term or long-term prognosis of patients with HF across the full spectrum of ejection fraction.

### Limitations

This study has several limitations. Firstly, other predisposing factors that may affect the BUN/Cr ratio, including the use of drugs such as corticosteroids and certain antibiotics, were not taken into consideration. Secondly, because this study is an observational study, other confounding factors affecting the results cannot be excluded, even after adjusted analysis. Finally, more studies are needed to further clarify the role of BUN/Cr in HF across the full spectrum of ejection fraction. Despite these limitations, our study emphasized that patients in the high BUN/Cr group had a poor long-term prognosis, and there was no significant correlation between high BUN/Cr with prognosis in patients with HFmrEF.

## Conclusion

The high BUN/Cr group is associated with the risk of poor prognosis of HFpEF, and is not lower than the predictive value of LVEF.

## *Supplemental Materials

For additional information, please click here.
